# Association of Serum Carotenoid Levels With Urinary Albumin Excretion in a General Japanese Population: The Yakumo Study

**DOI:** 10.2188/jea.JE20130058

**Published:** 2013-11-05

**Authors:** Koji Suzuki, Hisashi Honjo, Naohiro Ichino, Keisuke Osakabe, Keiko Sugimoto, Hiroya Yamada, Yasuhiro Kusuhara, Rika Watarai, Takeshi Hamajima, Nobuyuki Hamajima, Takashi Inoue

**Affiliations:** 1 Department of Public Health, Fujita Health University School of Health Sciences, Toyoake, Aichi, Japan; 1 藤田保健衛生大学医療科学部公衆衛生学教室; 2 Department of Clinical Acupuncture and Moxibustion, Meiji University of Integrative Medicine, Nantan, Kyoto, Japan; 2 明治国際医療大学鍼灸学部臨床鍼灸学教室; 3 Department of Clinical Physiology, Fujita Health University School of Health Sciences, Toyoake, Aichi, Japan; 3 藤田保健衛生大学医療科学部臨床生理学教室; 4 Department of Hygiene, Fujita Health University School of Medicine, Toyoake, Aichi, Japan; 4 藤田保健衛生大学医学部衛生学講座; 5 Department of Medical Zoology, Fujita Health University School of Health Sciences, Toyoake, Aichi, Japan; 5 藤田保健衛生大学医療科学部医動物学教室; 6 Department of Healthcare Administration, Nagoya University Graduate School of Medicine, Nagoya, Japan; 6 名古屋大学大学院医学系研究科健康社会医学専攻社会生命科学講座医療行政学/ヤング・リーダーズ・プログラム

**Keywords:** carotenoids, albuminuria, cross-sectional study

## Abstract

**Background:**

Albuminuria is a risk factor for not only nephropathy progression but also cardiovascular disease. Oxidative stress may have a role in the positive association between albuminuria and cardiovascular disease.

**Methods:**

This cross-sectional study investigated the associations of serum levels of carotenoids, which are dietary antioxidants, with albuminuria among 501 Japanese adults (198 men, mean age ± SD: 66.4 ± 10.0 years; 303 women, mean age ± SD: 65.4 ± 9.8 years) who attended a health examination. Serum levels of carotenoids were determined by high-performance liquid chromatography. Logistic regression analysis was used to estimate odds ratios (ORs) with 95% CIs for albuminuria after adjustment for age, body mass index, smoking habits, drinking habits, hypertension, diabetes mellitus, and dyslipidemia.

**Results:**

Prevalence of albuminuria was 15.4% among men and 18.1% among women. Among women with albuminuria, geometric mean serum levels of canthaxanthin, lycopene, β-carotene, total carotenes, and provitamin A were significantly lower than those of normoalbuminuric women. Adjusted ORs for albuminuria among women in the highest tertiles of serum β-carotene (OR, 0.45; 95% CI, 0.20–0.98) and provitamin A (OR, 0.45; 95% CI, 0.20–0.97) were significantly lower as compared with those for women in the lowest tertile. There were no associations between serum carotenoids and albuminuria in men.

**Conclusions:**

An increased level of serum provitamin A, especially serum β-carotene, was independently associated with lower risk of albuminuria among Japanese women.

## Abstract (ja)

**【背景】:**

アルブミン尿は腎症の進行のみならず心血管疾患のリスクファクターの一つである。アルブミン尿と心血管疾患との関連には酸化ストレスの関与が示唆されている。

**【方法】:**

我々は食事由来抗酸化物質であるカロテノイドの血清レベルとアルブミン尿との関連について住民健診受診者501人（男性198人、女性303人）を対象として横断解析を実施した。血清カロテノイド値は高速液体クロマトグラフィー法で分画測定した。ロジスティック回帰分析により、年齢、BMI、喫煙習慣、飲酒習慣、高血圧、糖尿病および脂質異常症を調整したアルブミン尿のオッズ比を算出した。

**【結果】:**

対象者のアルブミン尿の有病率は男性で15.4％、女性で18.1％であった。アルブミン尿の女性では、健常女性と比べ、血清カンタキサンチン、リコペン、β-カロテン、総カロテンおよびプロビタミンA値が有意に低い結果を得た。女性のアルブミン尿の調整オッズ比は、血清β-カロテン高値群（オッズ比：0.45、95%信頼区間：0.20-0.98）とプロビタミンA高値群（オッズ比：0.45、95%信頼区間：0.20-0.97）で有意に低かった。男性では血清カロテノイド値とアルブミン尿は有意な関連を認めなかった。

**【結論】:**

日本人女性では血清プロビタミンA、特にβ-カロテン値の上昇がアルブミン尿のリスク低下と関連することが示唆された。

## INTRODUCTION

Urinary albumin is a protein that increases with progression of glomerular damage and is used as a marker for early detection of nephropathy in people with diabetes. A small amount of albumin is filtered in glomeruli in healthy adults, and a urinary albumin level less than 30 mg/day is considered normal. Microalbuminuria is defined as excretion of 30 to 300 mg of albumin a day. The prevalence of microalbuminuria in Japan is approximately 13% to 20% and increases with age.^1^^,^^2^ Microalbuminuria is a risk factor for not only nephropathy progression but also cardiovascular disease (CVD).^3^^,^^4^ However, the mechanisms underlying the association between albuminuria and increased risk of CVD are not well understood.

High dietary intake of antioxidant vitamins was recently found to be associated with reduced risk of a decline in estimated glomerular filtration rate and microalbuminuria.^2^^,^^5^ The association of serum levels of carotenoids—antioxidants abundant in vegetables and fruits—with renal dysfunction and glomerular damage is controversial. Two studies^6^^,^^7^ reported that serum carotenoid level was inversely associated with microalbuminuria. In contrast, serum β-carotene level was significantly positively associated with serum creatinine level among a US population of non-Hispanic blacks.^8^

Serum carotenoid levels are markers of intakes of vegetables and fruits and have been reported to vary according to race/ethnicity, mainly due to differences in dietary habits.^9^ The prevalence of obesity, which is closely associated with albuminuria and serum carotenoid level, also differs by race/ethnicity.^10^ To our knowledge, no Japanese study has examined the association of serum carotenoid levels with albuminuria. We investigated these associations in a Japanese elderly population.

## METHODS

Since 1982, residents of the town of Yakumo in Hokkaido Prefecture, Japan aged 39 years or older have undergone routine health examinations. Cross-sectional and longitudinal studies of lifestyle-related diseases have enrolled individuals who participated in these health examinations. The present cross-sectional study is part of the ongoing Yakumo Study. A total of 514 residents (203 men and 311 women) attended a health examination in August 2011 and gave written informed consent. Of these, we excluded 3 people for whom urine samples were inadequate for measurement of albumin and creatinine. Thus, 511 adults (201 men and 310 women) were included in the current analysis. The protocol of this study was approved by the Ethics Committee of Fujita Health University (approval number 11-101).

Urinary spot samples and fasting serum samples were collected during the health examination. Urinary albumin was measured by immunonephelometry, and urinary creatinine was measured by an enzymatic method. Albuminuria was defined as a urinary albumin-to-creatinine ratio (UACR) of 30 mg or more albumin/g creatinine. Sera were separated from blood cells by centrifugation within 1 hour of sample collection. Serum levels of carotenoids, including zeaxanthin/lutein, canthaxanthin, β-cryptoxanthin, lycopene, α-carotene, and β-carotene, were measured by high-performance liquid chromatography, as previously reported.^11^ We calculated total carotenes as the sum of α-carotene, β-carotene, and lycopene; total xanthophylls as the sum of β-cryptoxanthin, zeaxanthin/lutein, canthaxanthin; and total provitamin A as the sum of α-carotene, β-carotene, and β-cryptoxanthin. Total carotenoids were calculated as total carotenes plus total xanthophylls. Other biochemical analyses of blood were performed using autoanalyzers in the laboratory at Yakumo General Hospital.

Trained nurses administered a questionnaire on health and daily lifestyle habits, including smoking (current smoker, ex-smoker, or nonsmoker), alcohol consumption (regular drinker, ex-drinker, or nondrinker), menopausal status (yes or no), and history of major illness. Anthropometric indices (height and weight) and blood pressure were measured during the health examination. Body mass index (BMI) was calculated as body weight (kg) divided by height (m) squared.

Hypertension was defined as a systolic blood pressure greater than 140 mm Hg and/or a diastolic blood pressure greater than 90 mm Hg (based on guidelines from the Japanese Society of Hypertension)^12^ or use of antihypertensive medications. Diabetes mellitus was defined as a fasting plasma glucose level of 126 mg/dl or higher and/or a glycated hemoglobin (HbA1c) value of 6.5% or higher and/or use of medication for diabetes. Japan Diabetes Society (JDS) HbA1c values (%) were converted to the equivalent National Glycohemoglobin Standardization Program (NGSP) values (%) by using the formula HbA1c (NGSP) = HbA1c (JDS) + 0.4%.^13^ Dyslipidemia was defined as a triglyceride level of 150 mg/dl or higher, a high-density lipoprotein cholesterol (HDL-C) level of less than 40 mg/dl, or a low-density lipoprotein cholesterol (LDL-C) level of 140 mg/dl or higher (based on the guidelines of the Japan Atherosclerosis Society),^14^ or use of antidyslipidemic drugs.

All statistical analyses were conducted using JMP version 10.0 software (SAS Institute, Cary, NC, USA). Because serum levels of carotenoids and UACR are distributed logarithmically, we used logarithms of these data for the analyses. Continuous variables were compared using the *t* test, and categorical variables were compared by the χ^2^ test. Logistic regression analysis was used to estimate odds ratios (ORs) with 95% CIs for albuminuria adjusted for potential confounders. We included age, BMI, smoking habits, drinking habits, hypertension, diabetes mellitus, and dyslipidemia as potential confounders. Further adjustment for menopausal status was performed in women. A *P* value of less than 0.05 was considered statistically significant.

## RESULTS

The characteristics of participants with and without albuminuria are presented in Table 1. There were 31 men (15.4%) and 56 women (18.1%) with albuminuria. Of these, 3 men and 5 women had macroalbuminuria (>300 mg/g creatinine). Mean age, systolic blood pressure, serum glucose, and HbA1c were significantly higher in both men and women with albuminuria than in those without albuminuria. As compared with normoalbuminuric men, BMI was significantly higher in men with albuminuria. Diastolic blood pressure was significantly higher among women with albuminuria than among normoalbuminuric women.

**Table 1.** Characteristics of the study subjects according to urinary albumin-to-creatinine (Cre) ratio

			Men			Women		
				
			Normoalbuminuria	Albuminuria	*P*	Normoalbuminuria	Albuminuria	*P*
			(<30 mg/g Cre)	(≥30 mg/g Cre)		(<30 mg/g Cre)	(≥30 mg/g Cre)	
No.			170	31		254	56	
		(%)	(84.6)	(15.4)		(81.9)	(18.1)	
Age^a^		(y)	65.7 ± 9.9	70.2 ± 9.9	0.022^c^	64.6 ± 9.4	69.0 ± 10.8	0.003^c^
Body mass index^a^		(kg/m^2^)	23.9 ± 3.0	25.0 ± 3.3	0.049^c^	23.3 ± 3.2	24.1 ± 4.6	0.128^c^
Systolic blood pressure^a^		(mm Hg)	131.6 ± 16.0	142.5 ± 18.0	<0.001^c^	130.6 ± 16.4	143.1 ± 18.1	<0.001^c^
Diastolic blood pressure^a^		(mm Hg)	79.1 ± 9.9	82.2 ± 13.2	0.133^c^	77.4 ± 8.3	81.4 ± 9.6	0.002^c^
Glucose^a^		(mg/dL)	91.5 ± 14.2	102.1 ± 30.6	0.003^c^	87.5 ± 13.1	97.0 ± 20.9	<0.001^c^
Hemoglobin A1c (NGSP)^a^		(%)	5.6 ± 0.4	6.1 ± 0.9	<0.001^c^	5.6 ± 0.6	5.8 ± 0.8	0.005^c^
LDL cholesterol^a^		(mg/dL)	122.1 ± 31.0	125.0 ± 22.8	0.625^c^	128.6 ± 31.2	122.8 ± 30.4	0.211^c^
HDL cholesterol^a^		(mg/dL)	55.7 ± 12.3	55.5 ± 14.9	0.930^c^	65.2 ± 13.4	65.0 ± 14.2	0.916^c^
Triglyceride^b^		(mg/dL)	104.4 (76.8–139.3)	121.5 (92.0–165.0)	0.099^c^	89.3 (67.0–118.3)	94.4 (70.8–133.0)	0.377^c^
Smoking	Non	*n*	46	7	0.731^d^	209	47	0.951^d^
		(%)	(27.0)	(22.6)		(82.3)	(83.9)	
	Ex	*n*	96	19		26	5	
		(%)	(56.5)	(61.3)		(10.2)	(8.9)	
	Current	*n*	28	5		19	4	
		(%)	(16.5)	(16.1)		(7.5)	(7.2)	
Drinking	Non	*n*	47	13	0.123^d^	185	41	0.990^d^
		(%)	(27.6)	(41.9)		(72.8)	(73.2)	
	Ex	*n*	12	0		4	1	
		(%)	(7.1)	(0.0)		(1.6)	(1.8)	
	Current	*n*	111	18		65	14	
		(%)	(65.3)	(58.1)		(25.6)	(25.0)	

Abbreviations: LDL, low-density lipoprotein; HDL, high-density lipoprotein.

^a^Data are expressed as mean ± SD.

^b^Data are expressed as geometric mean (interquartile range).

^c^*t* test.

^d^χ^2^ test.

Hemoglobin A1c (%) was converted to the National Glycohemoglobin Standardization Program (NGSP) equivalent value using the formula: hemoglobin A1c (NGSP) = hemoglobin A1c (Japan Diabetes Society) + 0.4%.

Table 2 shows the geometric mean serum levels of carotenoids according to UACR. Among women, the geometric mean serum levels of canthaxanthin, lycopene, β-carotene, total carotenes, and provitamin A were significantly lower in those with albuminuria than in normoalbuminuric women. There were no significant differences in serum carotenoid levels between men with and without albuminuria.

**Table 2.** Comparison of serum carotenoid levels according to urinary albumin-to-creatinine ratio

		Men			Women		
			
		Normoalbuminuria	Albuminuria	*P*^a^	Normoalbuminuria	Albuminuria	*P*^a^
		(<30 mg/g Cre)	(≥30 mg/g Cre)		(<30 mg/g Cre)	(≥30 mg/g Cre)	
Zeaxanthin/lutein	(µmol/l)	1.195 (0.901–1.719)	1.177 (0.874–1.612)	0.855	1.467 (1.220–1.979)	1.437 (1.156–1.847)	0.684
Canthaxanthin	(µmol/l)	0.054 (0.040–0.073)	0.049 (0.034–0.075)	0.309	0.065 (0.048–0.089)	0.053 (0.036–0.078)	0.003
β-Cryptoxanthin	(µmol/l)	0.180 (0.111–0.263)	0.176 (0.122–0.270)	0.861	0.321 (0.227–0.456)	0.286 (0.194–0.419)	0.153
Lycopene	(µmol/l)	0.507 (0.336–0.817)	0.442 (0.274–0.837)	0.323	0.734 (0.500–1.138)	0.603 (0.390–0.978)	0.040
α-Carotene	(µmol/l)	0.182 (0.112–0.279)	0.169 (0.098–0.267)	0.601	0.277 (0.184–0.427)	0.246 (0.145–0.470)	0.215
β-Carotene	(µmol/l)	0.628 (0.342–1.180)	0.653 (0.388–1.241)	0.810	1.397 (0.966–2.158)	1.136 (0.833–1.861)	0.025
Total carotenes	(µmol/l)	1.431 (0.936–2.189)	1.364 (0.927–2.414)	0.705	2.537 (1.812–3.661)	2.134 (1.472–3.256)	0.026
Total xanthophylls	(µmol/l)	1.460 (1.132–2.055)	1.426 (1.064–1.804)	0.774	1.892 (1.530–2.486)	1.820 (1.457–2.372)	0.429
Provitamin A	(µmol/l)	1.043 (0.638–1.765)	1.051 (0.637–1.802)	0.957	2.071 (1.511–3.045)	1.742 (1.275–2.744)	0.032
Total carotenoids	(µmol/l)	2.985 (2.170–4.253)	2.881 (2.078–4.266)	0.705	4.519 (3.490–6.015)	4.065 (3.062–5.532)	0.068

Data are expressed as geometric mean (interquartile range).

^a^*t* test.

Table 3 shows multivariate-adjusted ORs and 95% CIs for albuminuria by tertile of serum carotenoids. Among women, ORs for albuminuria among women in the highest tertiles of serum β-carotene (OR, 0.45; 95% CI, 0.20–0.98) and provitamin A (OR, 0.45; 95% CI, 0.20–0.98) were significantly lower than for those in the lowest tertiles. However, there were no significant associations of serum carotenoids with albuminuria in men. Further adjustment for menopausal status in women did not materially change any of the findings in Table 3 (data not shown).

**Table 3.** Adjusted^a^ odds ratios (ORs) and 95% CIs for albuminuria according to tertile of serum carotenoid level by sex

		Men				Women			
			
		Low	Middle	High	*P* for trend	Low	Middle	High	*P* for trend
Zeaxanthin/lutein	Albuminuria/Total (%)	12/67 (17.9)	10/67 (14.9)	9/67 (13.4)		19/103 (18.5)	21/104 (20.2)	16/103 (15.5)	
	OR (95%CI)	1.00	0.78 (0.28–2.13)	0.64 (0.21–1.86)	0.412	1.00	0.97 (0.46–2.05)	0.71 (0.32–1.57)	0.405
Canthaxanthin	Albuminuria/Total (%)	14/67 (20.9)	7/67 (10.5)	10/67 (14.9)		28/103 (27.2)	14/104 (13.5)	14/103 (13.6)	
	OR (95%CI)	1.00	0.33 (0.10–1.01)	0.76 (0.26–2.17)	0.549	1.00	0.53 (0.24–1.12)	0.57 (0.26–1.25)	0.132
β-Cryptoxanthin	Albuminuria/Total (%)	9/67 (13.4)	13/67 (19.4)	9/67 (13.4)		23/103 (22.3)	18/104 (17.3)	15/103 (14.6)	
	OR (95%CI)	1.00	1.77 (0.61–5.30)	0.96 (0.31–2.97)	0.925	1.00	0.70 (0.33–1.47)	0.64 (0.29–1.37)	0.242
Lycopene	Albuminuria/Total (%)	13/67 (19.4)	9/67 (13.4)	9/67 (13.4)		28/103 (24.3)	17/104 (16.4)	14/103 (13.6)	
	OR (95%CI)	1.00	0.59 (0.20–1.64)	0.65 (0.22–1.80)	0.390	1.00	0.72 (0.34–1.50)	0.68 (0.31–1.46)	0.303
α-Carotene	Albuminuria/Total (%)	11/67 (16.4)	8/67 (11.9)	12/67 (17.9)		26/103 (25.2)	18/103 (17.5)	12/104 (11.5)	
	OR (95%CI)	1.00	0.72 (0.24–2.08)	1.28 (0.44–3.76)	0.694	1.00	0.82 (0.38–1.72)	0.56 (0.25–1.23)	0.155
β-Carotene	Albuminuria/Total (%)	9/67 (13.4)	11/67 (16.4)	11/67 (16.4)		26/103 (25.2)	16/104 (15.4)	14/103 (13.6)	
	OR (95%CI)	1.00	1.46 (0.50–4.41)	1.38 (0.44–4.59)	0.584	1.00	0.56 (0.26–1.18)	0.45 (0.20–0.98)	0.043
Total carotenes	Albuminuria/Total (%)	11/67 (16.4)	9/67 (13.4)	11/67 (16.4)		26/103 (25.2)	16/104 (15.4)	14/104 (13.6)	
	OR (95%CI)	1.00	0.97 (0.34–2.75)	1.18 (0.40–3.49)	0.766	1.00	0.63 (0.30–1.32)	0.56 (0.25–1.23)	0.135
Total xanthophylls	Albuminuria/Total (%)	12/67 (17.9)	12/67 (17.9)	7/67 (10.5)		21/103 (20.4)	18/104 (17.3)	17/103 (16.5)	
	OR (95%CI)	1.00	0.91 (0.34–2.44)	0.46 (0.14–1.44)	0.204	1.00	0.66 (0.30–1.44)	0.65 (0.29–1.43)	0.288
Provitamin A	Albuminuria/Total (%)	8/67 (11.9)	13/67 (19.4)	10/67 (14.9)		27/103 (26.2)	15/104 (14.4)	14/103 (13.6)	
	OR (95%CI)	1.00	1.74 (0.61–5.25)	1.39 (0.43–4.74)	0.562	1.00	0.49 (0.23–4.04)	0.45 (0.20–0.98)	0.037
Total carotenoids	Albuminuria/Total (%)	9/67 (13.4)	10/67 (14.9)	12/67 (17.9)		26/103 (25.2)	15/104 (14.4)	15/103 (14.6)	
	OR (95%CI)	1.00	1.09 (0.37–3.24)	1.49 (0.50–4.62)	0.471	1.00	0.57 (0.26–1.19)	0.59 (0.27–1.29)	0.159

^a^Adjusted for age, body mass index, smoking habit, drinking habit, hypertension, diabetes mellitus, and dyslipidemia.

## DISCUSSION

This is the first report to investigate the associations of serum carotenoid levels with albuminuria by sex in a Japanese population. We found that, in a Japanese elderly population, women with albuminuria had lower levels of serum provitamin A, especially β-carotene, as compared with normoalbuminuric women. This association remained statistically significant after adjustment for important confounders, including age, BMI, smoking habits, drinking habits, hypertension, diabetes mellitus, and dyslipidemia. Further adjustment for menopausal status in women did not materially alter the significant inverse association between serum carotenoids and albuminuria. These associations may be involved in the positive association of albuminuria with CVD risk.^3^^,^^4^ However, there were no associations of serum levels of carotenoids with albuminuria in men.

Our findings were consistent with those of 2 previous cross-sectional studies,^6^^,^^7^ which found independent associations between low levels of serum carotenoids and microalbuminuria, although the types of carotenoids in serum that were significantly associated with albuminuria were not completely consistent. In an Australian study, microalbuminuria was associated with lower plasma levels of α-carotene, β-cryptoxanthin, and lycopene in both sexes, and with lower plasma levels of β-carotene in men.^6^ Another study reported that serum β-cryptoxanthin, lutein/zeaxanthin, lycopene, and total carotenoids were inversely associated with microalbuminuria in a US population.^7^

It was reported that hypertensive patients with microalbuminuria had a lower level of reduced glutathione and a higher oxidized/reduced glutathione ratio as compared with normoalbuminuric hypertensive patients.^15^ Other biological markers of oxidative stress, such as plasma levels of malondialdehyde and protein carbonyl^16^ and urinary 8-hydroxydeoxyguanosine,^17^ were significantly positively correlated with microalbuminuria in people with type 2 diabetes. These findings suggest that albuminuria has a role in increased oxidative stress. Accordingly, people with albuminuria may have an oxidant–antioxidant imbalance and may thus need additional antioxidants to maintain proper oxidative balance.

Oxidative stress also has an important role in inflammation. High levels of reactive oxygen species cause activation of redox-sensitive genes and reaction with nitric oxide and affect arachidonic acid metabolism.^18^ This is believed to increase inflammation markers, lipid peroxidation, and endothelial dysfunction. A cross-sectional study reported that albumin/creatinine ratio was positively associated with biomarkers of inflammation, such as C-reactive protein, interleukin-6, and serum amyloid A.^19^ Oxidative stress and inflammation were suggested as possible causes of the association between albuminuria and CVD.

Carotenoids are potent quenchers of singlet molecular oxygen^20^ and also have an important role in decreasing activation of proinflammatory pathways, through quenching of free radicals.^21^ Circulating carotenoids are considered to be useful biomarkers of total dietary intake of vegetables and fruits.^22^ Epidemiologic studies reported that dietary intake of vegetables and fruits rich in carotenoids,^23^^,^^24^ and high serum levels of carotenoids,^25^^–^^27^ were associated with decreased CVD risk. The antioxidant and anti-inflammatory properties of carotenoids are believed to lower CVD risk. These properties of carotenoids may be also involved in the decreasing the risk of albuminuria. The pathogenesis and development of albuminuria and CVD have common background characteristics, which suggests the possibility of cardio–renal interaction. A previous cross-sectional study reported that higher intake of vegetable protein had a borderline inverse association with microalbuminuria.^5^ Dietary β-cryptoxanthin^2^ and a diet rich in whole grains, fruits, and vegetables^28^ were inversely associated with UACR. These findings suggest that a high intake of vegetables and fruits helps prevent microalbuminuria. However, we do not recommend the use of synthetic β-carotene supplements, because intervention studies^29^^,^^30^ have shown no beneficial effect of supplemental synthetic β-carotene on CVD risk. Since high doses of carotenoids may have a pro-oxidant effect, one possibility is that the effects of carotenoids in protecting against CVD disappear at the high doses used in supplementation studies. Supplementation with β-carotene had little effect on our results, because only 1 man and 3 women in this study used β-carotene supplements.

There were sex differences in the association of serum carotenoids with albuminuria in our study. Women with albuminuria had decreased levels of serum provitamin A as compared with normoalbuminuric women, but no significant associations were observed between serum carotenoids and albuminuria in men. The reason for this sex discrepancy is not clear. As compared with nonsmokers and nondrinkers, smokers and alcohol drinkers have reduced circulating levels of carotenoids.^31^^,^^32^ Also, cigarette smoking and alcohol drinking were significantly associated with albuminuria.^33^ Several studies in experimental animals and humans have shown that progression of renal function is influenced by gender,^34^^,^^35^ suggesting that sex steroid hormones may have a role in the pathogenesis of chronic kidney disease. We hypothesize that cigarette smoking, alcohol drinking, and hormonal factors have a considerable impact on the associations between serum carotenoids and albuminuria, especially in men. Moreover this study lacked adequate statistical power, especially in men, because the number of people with albuminuria was not large. Additional studies, with larger sample sizes, are required to clarify this discrepancy between sexes.

We were unable to examine causal relationships because of the cross-sectional design of the study. Chronic kidney disease is itself a state of low-grade inflammation.^36^ Inflammation may be an important cause of the increased oxidative stress observed in people with renal dysfunction. Prospective studies are needed to confirm a true causal relationship. Furthermore, the possibility of residual confounding cannot be completely ruled out, although confounding was appropriately adjusted for in our analyses.

In conclusion, an increased level of serum provitamin A, especially serum β-carotene, was associated with lower risk of albuminuria among women, after adjustment for possible confounding factors. These findings suggest that a diet rich in provitamin A could help prevent albuminuria, thereby reducing the risks of renal disease and CVD.

## ONLINE ONLY MATERIALS

Abstract in Japanese.
