# Supplementary material for: Association of Serum Carotenoid Levels With Urinary Albumin Excretion in a General Japanese Population: The Yakumo Study
Source: J Epidemiol. 2013 Nov 5;23(6):451–6. doi: 10.2188/jea.JE20130058 (PMC3834283; doi:10.2188/jea.JE20130058)
Supplement: Abstract in Japanese. [file je-23-451-s001.pdf]

一般住民における血清カロテノイド値と尿中アルブミン排泄量との関連：八雲スタディー

鈴木康司 1、本城久司 2、市野直浩 3、刑部恵介 3、杉本恵子 3、山田宏哉 4、楠原康弘 5、度會理佳 1、濱島　剛 1、濵嶋信之 6、井上　孝 1

1 藤田保健衛生大学医療科学部公衆衛生学教室
2 明治国際医療大学鍼灸学部臨床鍼灸学教室
3 藤田保健衛生大学医療科学部臨床生理学教室
4 藤田保健衛生大学医学部衛生学講座
5 藤田保健衛生大学医療科学部医動物学教室
6 名古屋大学大学院医学系研究科健康社会医学専攻社会生命科学講座医療行政学/ヤング・リーダーズ・プログラム

【背景】アルブミン尿は腎症の進行のみならず心血管疾患のリスクファクターの一つである。アルブミン尿と心血管疾患との関連には酸化ストレスの関与が示唆されている。
【方法】我々は食事由来抗酸化物質であるカロテノイドの血清レベルとアルブミン尿との関連について住民健診受診者 501 人（男性 198 人、女性 303 人）を対象として横断解析を実施した。血清カロテノイド値は高速液体クロマトグラフィー法で分画測定した。ロジスティック回帰分析により、年齢、BMI、喫煙習慣、飲酒習慣、高血圧、糖尿病および脂質異常症を調整したアルブミン尿のオッズ比を算出した。
【結果】 対象者のアルブミン尿の有病率は男性で 15.4%、女性で 18.1%であった。アルブミン尿の女性では、健常女性と比べ、血清カンタキサンチン、リコペン、β-カロテン、総カロテンおよびプロビタミン A 値が有意に低い結果を得た。女性のアルブミン尿の調整オッズ比は、血清β-カロテン高値群（オッズ比：0.45、95%信頼区間：0.20-0.98）とプロビタミン A 高値群（オッズ比：0.45、95%信頼区間：0.20-0.97）で有意に低かった。男性では血清カロテノイド値とアルブミン尿は有意な関連を認めなかった。
【結論】日本人女性では血清プロビタミン A、特にβ-カロテン値の上昇がアルブミン尿のリスク低下と関連することが示唆された。

キーワード: カロテノイド、アルブミン尿、横断研究
